# Abnormal Temporal Slowing on EEG Findings in Preclinical Alzheimer’s Disease Patients With the ApoE4 Allele: A Pilot Study

**DOI:** 10.7759/cureus.47852

**Published:** 2023-10-28

**Authors:** Nathan N Kim, Charissa Tan, Enze Ma, Selin Kutlu, Enrique Carrazana, Vajjhala Vimala, Jason Viereck, Kore Liow

**Affiliations:** 1 Neurology, John A. Burns School of Medicine (JABSOM), University of Hawaii, Honolulu, USA; 2 Brain Research, Innovation, & Translation Laboratory, Comprehensive Epilepsy Center & Video-EEG Epilepsy Monitoring Unit, Hawaii Pacific Neuroscience, Honolulu, USA; 3 Neurology, Hawaii Pacific Neuroscience, Honolulu, USA; 4 Brain Research, Innovation, & Translation Laboratory, Hawaii Pacific Neuroscience, Honolulu, USA

**Keywords:** preclinical, temporal slowing, electroencephalogram, dementia, apoe4, alzheimer's disease

## Abstract

Introduction: Currently, there are limited accessible and cost-effective biomarkers for preclinical Alzheimer’s disease (AD) patients. However, the apolipoprotein E (ApoE) polymorphic alleles can predict if someone is at high (e4), neutral (e3), or low (e2) genetic risk for developing AD. This study analyzed electroencephalogram (EEG) reports from individuals with various ApoE genotypes, aiming to identify EEG changes and patterns that could potentially serve as predictive markers for preclinical AD progression.

Methods: Participants aged 64-78 were selected from the patient database at an outpatient neurology clinic. Genotype studies were performed to determine ApoE status, followed by EEG analysis to identify any apparent trends. A case-control design was used, categorizing participants into cases (e2e3, e2e4, e3e4, e4e4) and controls (e3e3). EEG recordings were compared between the groups to identify potential differences in EEG characteristics, including abnormal temporal slowing, frequency, and ApoE genotype association.

Results: Among 43 participants, 49% demonstrated evidence of abnormal temporal slowing on EEG. Of these, 48% displayed focal left temporal slowing, and 52% displayed bilateral temporal slowing. The right-sided temporal slowing was not observed. Among participants with abnormal slowing, 95% exhibited theta frequency (4-8 Hz) slowing, while only 4.8% displayed delta frequency (0-4 Hz) slowing. Among participants with the ApoE4 allele, 61.5% demonstrated evidence of abnormal slowing, compared to 43.3% without it. Furthermore, the presence of an ApoE4 allele was associated with a significantly higher proportion of males (54%) compared to those without it (13%) (p=0.009).

Conclusions: Although we did not find a statistically significant difference in temporal EEG slowing among different ApoE genotypes, our findings suggest a potential association between temporal slowing on EEG and the presence of an ApoE4 allele in individuals with preclinical AD. These observations highlight the need for further exploration into the potential influence of the ApoE4 allele on EEG findings and the utility of EEG as a complementary diagnostic tool for AD. Longitudinal studies with large sample sizes are needed to establish the precise relationship between EEG patterns, ApoE genotypes, and AD progression.

## Introduction

Alzheimer’s disease (AD) is a progressive neurodegenerative disease and the leading cause of dementia. In 2018, around 50 million individuals were affected, with projections indicating that this number could surpass 150 million by 2050 [1,2]. AD is characterized by an insidious onset of cognitive decline, impaired functional status, and abnormal behavioral changes [3,4]. Diagnosis of AD is based on clinical criteria, which are supported by neuropsychologic assessments, cognitive exams such as the mini-mental state examination (MMSE), amyloid positron emission tomography (PET) scans, magnetic resonance imaging (MRI), or cerebrospinal fluid (CSF) analysis [3-6]. However, these tests may not always be readily available, and CSF sampling is an invasive procedure. Furthermore, the memory loss associated with AD is largely permanent by the time of diagnosis.

The pathophysiology of AD is complex and multifactorial. One theory suggests that it involves the accumulation of proteins beta-amyloid and hyperphosphorylated tau in the brain, leading to neuronal cell death [3]. Studies suggest that molecular changes within the brain may precede measurable cognitive impairment, emphasizing the potential for delayed diagnosis decades after disease onset [5,7]. However, there are currently no reliable tests or biomarkers to diagnose preclinical AD apart from CSF or amyloid PET imaging [3]. Therefore, key risk factors must be considered when evaluating patients. One of the most important and widely studied risk factors is the apolipoprotein E (ApoE) gene [8]. This genetic factor has three alleles that each exert varying influences on an individual’s risk for AD: ApoE2 decreases risk, ApoE3 is neutral, and ApoE4 increases risk [8-11]. Additional factors such as age, family history, socioeconomic status, and positive CSF amyloid levels also contribute to the likelihood of developing AD [3,9].

Despite extensive research, there is no curative treatment for AD. However, early detection and diagnosis allow for the initiation of disease-modifying therapies to slow the progression of the disease. Therefore, early detection, timely diagnosis, and appropriate management of AD are imperative [6,12]. Previous studies have demonstrated that EEG changes can be observed in AD patients, especially those with significant risk factors. EEG measures neuronal activity within the brain, offering the potential to detect alterations in electrical potentials before structural changes occur [13]. Studies investigating EEG patterns in AD patients reveal slowing EEG rhythms, with risk factors such as ApoE4 accentuating these results [3-4,14-15]. However, there is limited research on EEG findings specifically related to ApoE genotypes in individuals with preclinical AD. Therefore, this pilot study aims to determine if there are specific EEG characteristics in preclinical AD patients with known ApoE status by comparing the EEG recordings across different ApoE genotypes. Comparing EEGs of high-ris­­k and low-risk preclinical AD patients may reveal patterns supporting the use of EEG as a marker for detecting AD progression, potentially reducing the need for further costly and invasive diagnostic procedures.

## Materials and methods

This study was sanctioned by the University of Hawaii Institutional Review Board (protocol number: 2019-00054), and written informed consent was obtained from all subjects as appropriate.

Study design 

A case-control design was used to investigate specific EEG findings in preclinical AD patients with known ApoE status. Participants were categorized into two groups: cases and controls. The control group consisted of individuals with an e3e3 ApoE genotype, while the other ApoE genotype groups (e2e3, e2e4, e3e4, e4e4) constituted the cases. A comprehensive analysis was conducted to evaluate the impact of different ApoE genotypes on participant characteristics. This analysis aimed to identify potential differences in gender distribution, age, and the presence of temporal slowing on EEG among participants with different ApoE genotypes. Statistical analysis was made using Fisher's exact test and the Kruskal-Wallis rank sum test. The significance level was set at p<0.05. All calculations were performed in R (version 4.3.1; R Development Core Team, Vienna, Austria).

To investigate the relationship between the presence or absence of ApoE4 and its association with temporal slowing on EEG, a separate analysis was conducted. Statistical analysis was made using Fisher's exact test to compare the presence of temporal slowing on EEG between participants with and without ApoE4. To examine the differences in gender distribution and age between participants with and without ApoE4, Fisher's exact test and Welch's two-sample t-test were used. A separate subgroup analysis was performed for individuals who demonstrated bilateral slowing. Fisher’s exact test was used to evaluate differences in ApoE4 presence between individuals with independent and synchronous bilateral slowing. The significance level was set at p<0.05. All calculations were performed in R (version 4.3.1).

Subjects 

Participants (n=43) were recruited from a larger cohort of patients (n=127) screened for ApoE status at an outpatient neurology clinic from 2019 to 2022. Individuals were identified as having preclinical AD using specific inclusion and exclusion criteria. Inclusion criteria required participants to be 18 years of age or older, display no clinical signs or symptoms of AD, and have at least one marker of AD pathology. Exclusion criteria encompassed a prior diagnosis of either AD or mild cognitive impairment and a history of major structural brain disease, stroke, transient ischemic attack, traumatic brain injury, or seizures. To ensure cognitive health, a minimum MMSE score of 28 was required. ApoE genotype was determined through genetic testing using standard molecular biology techniques.

EEG recordings

EEG recordings were conducted at an outpatient neurology clinic using the standard 10-20 measuring system and gold disc electrodes affixed with Ten20 conductive paste (Weaver and Company, Norwalk, CA). The Cadwell Arc recording hardware and software were utilized, along with 22 standard scalp leads and a two-lead, single-channel electrocardiogram. Scalp electrode impedances were reduced to 10 kΩ or lower. The primary montage used was the "longitudinal anatomic bipolar" with the option for rederivation to a different montage. Patients were reclined comfortably during the recordings, which lasted between 25 and 30 minutes.

Reactivity was measured with eye opening and closing while patients were awake and alert. Photic stimulation was performed at rates of 1, 3, 6, 12, 15, 18, 20, and 30 Hz, for 10 seconds each, with 10 seconds in between. Hyperventilation was performed for three minutes unless medically contraindicated. The remaining study time was dedicated to quiet recording to facilitate drowsiness or sleep. EEG reports were analyzed using visual analysis and manual counting by a fellowship-trained neurophysiologist.

In this study, "slowing" was defined as a reduction in the frequency of the brain wave pattern waveforms compared to the expected normal range for the participants' age and cognitive function. In this study, we specifically looked at slowing in the temporal region. Slowing was categorized into two frequency ranges: delta (0-4 Hz) and theta (4-8 Hz). Slowing was also categorized as intermittent or continuous. Slowing was further characterized as focal or bilateral. Bilateral slowing was categorized as independent or synchronous. Additionally, we recorded whether participants were able to attain sleep during the EEG study, the presence of findings in wakefulness, drowsiness, or both and the presence of epileptiform changes.

## Results

Patient characteristics

This study involved 43 participants (Table 1), consisting of 11 (26%) males and 32 (74%) females. Ages ranged from 64 to 78 years with a mean age of 70.7 years. Participants were stratified according to their ApoE genotype, with the following distribution: e2e3 (n=3), e2e4 (n=1), e3e3 (n=27), e3e4 (n=8), and e4e4 (n=4). All participants had MMSE scores of 28 or higher.

**Table 1:** Patient Characteristics

	ApoE						
Characteristics	Overall, N=43^1^	e2e3, N=3^1^	e2e4, N=1^1^	e3e3, N=27^1^	e3e4, N=8^1^	e4e4, N=4^1^	p-value^2^
Male	11 (26%)	0 (0%)	0 (0%)	4 (15%)	5 (63%)	2 (50%)	0.028
Mean Age ± SD (years)	70.7 (4.5)	71.3 (3.2)	74.0 (NA)	70.9 (4.6)	70.3 (4.4)	68.3 (5.7)	0.816
Temporal Slowing	21 (49%)	2 (67%)	1 (100%)	11 (41%)	5 (63%)	2 (50%)	0.585
^1^n (%); Mean (SD)							
^2^Fisher's exact test; Kruskal-Wallis rank sum test							

EEG features

Among the 43 participants, 21 (49%) displayed evidence of abnormal temporal slowing on EEG (Table 2). The percentage of participants with abnormal slowing in each genotype group was as follows: e2e3 (67%), e2e4 (100%), e3e3 (41%), e3e4 (63%), and e4e4 (50%). The difference between these groups was not statistically significant (p=0.585). Among individuals who displayed temporal slowing, 11 (52%) exhibited focal left-sided temporal slowing, and 10 (48%) displayed bilateral temporal slowing. No participants demonstrated evidence of right-sided temporal slowing. Among participants with bilateral slowing, five (50%) showed independent bilateral slowing (Table 3), while five (50%) demonstrated synchronous bilateral slowing. All participants with abnormal slowing displayed intermittent slowing, with one (4.8%) displaying slowing in the delta (0-4 Hz) frequency range and 20 (95%) displaying slowing in the theta (4-8 Hz) frequency range. During the EEG study, 39 participants (90.7%) achieved sleep at some point. Findings in wakefulness were observed in two participants (9.5%), and findings in drowsiness were observed in four participants (19%). One participant (4.8%) displayed findings in both wakefulness and drowsiness. Among participants with abnormal slowing, 20 (95.2%) were able to attain sleep during the EEG study. No epileptiform changes were observed for any of the participants.

**Table 2:** EEG Features

	ApoE4 Status			
Characteristics	Overall, N=21^1^	ApoE4 Absent, N=13^1^	ApoE4 Present, N=8^1^	p-value^2^
Temporal Slowing				0.659
Bilateral	10 (48%)	7 (54%)	3 (38%)	
Focal Left	11 (52%)	6 (46%)	5 (63%)	
Frequency				0.381
Delta (0-4 Hz)	1 (4.8%)	0 (0%)	1 (13%)	
Theta (4-8 Hz)	20 (95%)	13 (100%)	7 (88%)	
^1^n (%)				
^2^Fisher's exact test				

**Table 3:** Bilateral Slowing Subgroup Analysis

	Bilateral Slowing			
Characteristics	Overall, N=10^1^	Bilateral Slowing Absent, N=7^1^	Bilateral Slowing Present, N=3^1^	p-value^2^
Bilateral Type				>0.999
Independent	5 (50%)	3 (43%)	2 (67%)	
Synchronous	5 (50%)	4 (57%)	1 (33%)	
^1^n (%)				
^2^Fisher's exact test				

EEG slowing and ApoE4 presence

Among individuals without an ApoE4 allele present in their genotype (Table 4), 13% were male, while, among those with an ApoE4, 54% were male (p=0.009). The average age of all participants was 70.7 years. The average age for participants without an ApoE4 was 71.0 years, and, for participants with an ApoE4, it was 69.9 years (p=0.502). Among all participants, 49% demonstrated evidence of temporal slowing on EEG. Among those without an ApoE4, 43% had temporal slowing, and, among those with an ApoE4, 62% had temporal slowing (p=0.332).

**Table 4:** ApoE4 Present Versus ApoE4 Absent

	ApoE4 Status			
Characteristics	Overall, N=43^1^	ApoE4 Absent, N=30^1^	ApoE4 Present, N=13^1^	p-value^2^
Male	11 (26%)	4 (13%)	7 (54%)	0.009
Age	70.7 (4.5)	71.0 (4.4)	69.9 (4.7)	0.502
Temporal Slowing	21 (49%)	13 (43%)	8 (62%)	0.332
^1^n (%); Mean (SD)				
^2^Fisher's exact test; Welch two-sample t-test				

## Discussion

The findings of this study highlight the association between ApoE genotypes and EEG findings in individuals with preclinical AD. Although the association between ApoE4 and abnormal temporal slowing did not reach statistical significance, the trends suggest that individuals carrying an ApoE4 allele may be more likely to exhibit abnormal temporal slowing on EEG. Additionally, when comparing individuals with e2e3 and e2e4 genotypes, those with the e2e4 genotype were more likely to display abnormal temporal slowing compared to e2e3 individuals, further supporting the existence of a relationship between the ApoE4 allele and temporal slowing on EEG.

Several studies have investigated EEG changes in individuals with AD. They report that certain EEG abnormalities are linked to cognitive deficits in AD patients at different stages of the disease, and EEG has promise as a potential tool to predict future neurodegeneration in AD patients [16,17]. One study successfully used EEG to characterize AD patients across the disease spectrum, from preclinical to advanced stages [18]. Altogether, these findings emphasize the potential utility of EEG as a non-invasive marker for monitoring neurodegenerative changes in AD. Further research is needed to fully elucidate its clinical value in predicting disease progression and guiding therapeutic interventions at various stages of the disease.

The relationship between EEG slowing in the theta-delta region and AD is well-established [3]. Individuals with AD display a shift towards lower frequencies and decreased coherence of fast rhythms, along with increased activity in the delta and theta frequency bands and decreased activity in the alpha and beta bands on EEG [3]. Although our study exclusively included individuals with preclinical AD, we observed a similar shift toward delta and theta frequencies among individuals displaying temporal slowing. Interestingly, only one participant demonstrated slowing in the delta frequency range, while the majority of individuals with temporal slowing exhibited slowing in the theta frequency range. It is unknown if the severity of AD affects an individual’s propensity to display slowing in the delta or theta frequency range. Therefore, investigating the prevalence of theta slowing and delta slowing between individuals with preclinical AD, MCI, and AD could be an interesting area for future EEG research.

Interestingly, a smaller proportion of individuals with an e4e4 genotype displayed abnormal temporal slowing compared to those with the e3e4 genotype. Homozygous ApoE4 genotype (e4e4) has been linked to reduced EEG functional connectivity in various brain regions of AD patients compared to heterozygous ApoE4 carriers and controls [14]. Moreover, there is evidence of a significant dosage effect, with each additional e4 allele accelerating the onset of AD to an earlier age [11]. Therefore, we expected a greater proportion of e4e4 patients to demonstrate temporal slowing compared to e3e4 individuals. However, our findings suggest that the absolute number of ApoE4 alleles a person carries may not directly correlate with the presence of temporal slowing on EEG.

The ApoE4 genotype is thought to contribute to neurodegenerative processes, negatively impacting cortical activity in both healthy individuals and those with AD [19]. Since the ApoE4 allele is a known genetic risk factor for AD [20], individuals with the ApoE4 allele may have an elevated susceptibility to the neurodegenerative changes associated with abnormal EEG slowing. These EEG changes are believed to reflect brain functional deficits related to the ApoE4 allele [21]. For example, one study observed reduced EEG measures of global synchronization in theta and beta bands in patients on a biomarker-verified AD continuum with the ApoE4 genotype [21]. Although our study focused on temporal slowing, it is crucial to consider other EEG features when evaluating individuals at risk for AD. For instance, one study reported a relationship between the presence of ApoE4 and increased alpha rhythm slowing [22]. Considering the multitude of EEG factors, further research is needed to comprehensively assess the utility of EEG in preclinical AD patients.

The statistically significant difference in the proportion of males between individuals with and without an ApoE4 allele in their genotype was an interesting finding. While unintentional, this discrepancy could have potentially influenced the study outcomes, as AD can present differently in males and females [23]. One study found that ApoE4 has a more significant impact on AD risk in females compared to males [24]. Therefore, the difference in male proportion between the two groups may have influenced our findings. Future studies should consider controlling for potential confounding factors such as sex and aim to include a more balanced representation of sexes to improve the generalizability and reliability of the results.

There are several limitations of this study. Notably, the relatively small sample size for certain genotypes may have affected the statistical power of the analysis. Therefore, drawing definitive conclusions from these specific subgroups should be done with caution. For instance, the e2e4 genotype was represented by only one participant, and the e4e4 genotype was represented by only four participants. A larger sample size would have allowed for more robust statistical analyses and increased generalizability. However, given that this pilot study serves as a preliminary exploration, a smaller sample size was appropriate. Another limitation was that the EEG analyses were made visually and, therefore, subject to the reader’s interpretation. A standardized approach using quantitative EEG analysis could provide more objective and reliable results. While the practicality of EEG usage is advantageous, relying solely on visual analysis can introduce potential biases. Future studies should consider utilizing both visual EEG analysis and quantitative EEG analysis to enhance the validity and reliability of their findings. Finally, accurately identifying individuals at the preclinical stage of AD versus those at more advanced stages of AD was challenging due to the subjective nature of the diagnosis, which can vary among different providers. It is possible that some individuals included in the study were at more advanced stages of the disease, potentially influencing the study results.

## Conclusions

Biomarkers for timely detection and diagnosis of AD before the onset of permanent memory loss are crucial. Although no statistically significant relationship was observed between temporal EEG slowing and the ApoE4 allele in individuals with normal cognitive function, the trends suggest that the ApoE4 allele may influence EEG findings. Future investigations are needed to accurately assess the predictive value of EEG as a readily available, noninvasive, and inexpensive tool for screening and identifying individuals at high risk of developing AD. These studies should examine longitudinal data in a larger patient population with various ApoE genotypes and examine the appearance of EEG temporal slowing at different stages as patients progress from preclinical, to mild, to severe AD. Altogether, these findings could enhance our understanding of the pathophysiology of disease progression and the potential window of opportunity for intervention with disease-modifying therapies.
